# Binocular Summation and Other Forms of Non-Dominant Eye Contribution in Individuals with Strabismic Amblyopia during Habitual Viewing

**DOI:** 10.1371/journal.pone.0077871

**Published:** 2013-10-29

**Authors:** Brendan T. Barrett, Gurvinder K. Panesar, Andrew J. Scally, Ian E. Pacey

**Affiliations:** 1 Bradford School of Optometry & Vision Science, University of Bradford, Bradford, West Yorkshire, United Kingdom; 2 School of Health Studies, University of Bradford, Bradford, West Yorkshire, United Kingdom; Harvard Medical School, United States of America

## Abstract

**Background:**

Adults with amblyopia (‘lazy eye’), long-standing strabismus (ocular misalignment) or both typically do not experience visual symptoms because the signal from weaker eye is given less weight than the signal from its fellow. Here we examine the contribution of the weaker eye of individuals with strabismus and amblyopia with both eyes open and with the deviating eye in its anomalous motor position.

**Methodology/Results:**

The task consisted of a blue-on-yellow detection task along a horizontal line across the central 50 degrees of the visual field. We compare the results obtained in ten individuals with strabismic amblyopia with ten visual normals. At each field location in each participant, we examined how the sensitivity exhibited under binocular conditions compared with sensitivity from four predictions, (i) a model of binocular summation, (ii) the average of the monocular sensitivities, (iii) dominant-eye sensitivity or (iv) non-dominant-eye sensitivity. The proportion of field locations for which the binocular summation model provided the best description of binocular sensitivity was similar in normals (50.6%) and amblyopes (48.2%). Average monocular sensitivity matched binocular sensitivity in 14.1% of amblyopes’ field locations compared to 8.8% of normals’. Dominant-eye sensitivity explained sensitivity at 27.1% of field locations in amblyopes but 21.2% in normals. Non-dominant-eye sensitivity explained sensitivity at 10.6% of field locations in amblyopes but 19.4% in normals. Binocular summation provided the best description of the sensitivity profile in 6/10 amblyopes compared to 7/10 of normals. In three amblyopes, dominant-eye sensitivity most closely reflected binocular sensitivity (compared to two normals) and in the remaining amblyope, binocular sensitivity approximated to an average of the monocular sensitivities.

**Conclusions:**

Our results suggest a strong positive contribution in habitual viewing from the non-dominant eye in strabismic amblyopes. This is consistent with evidence from other sources that binocular mechanisms are frequently intact in strabismic and amblyopic individuals.

## Introduction

The term ‘strabismus’ refers to the condition in which only one of the eyes is directed at the object of interest. Such misalignment of the visual axes has an extremely high prevalence (∼5% of the general population, [1], [2]) and a range of possible causes and aetiologies [3]–[5]. Although strabismus can be acquired in adulthood, typically it presents as a developmental disorder of vision in early childhood [2], coinciding with the critical period for visual development [6].

In addition to the negative psychosocial impact of living with strabismus which can be significant (e.g. reported difficulties making eye contact, [7]), there are potentially significant consequences for the individual’s visual capabilities. The presence of strabismus is associated with either no clinically-measurable stereopsis (recovery of depth information based upon the disparate views of the right and left eyes) or stereopsis that is markedly degraded [2]. Loss of stereopsis has important functional consequences in everyday activities, in particular for fine motor tasks [8], [9]. Alongside the psychosocial and functional consequences of strabismus, the presence of strabismus in early life is a known risk factor for amblyopia [10], another developmental disorder of vision that is typically unilateral and in which visual acuity is subnormal despite optimal optical correction and an eye /visual system that is apparently healthy [11]. Although amblyopia can exist without strabismus and strabismus can exist without amblyopia, these two conditions frequently co-exist; for example, approximately two-thirds of amblyopic individuals also exhibit strabismus [12]. This study is concerned with an examination of visual function in individuals with both strabismus and amblyopia.

Strabismus creates at least two problems for the visual system and these are classically termed “confusion” and “diplopia” ([2], [11], [12], also see our previous paper for description and demonstration, [13]). Amblyopia presents the visual system with an additional problem; since the percept in the affected eye is not clear, there is the possibility of simultaneous, super-imposed perception of one clear and one blurred/distorted image. The visual system is widely believed to deal with the problems caused by strabismus and amblyopia using a mechanism that involves the active *suppression* of the image in the deviating/amblyopic eye ([11], [12], [14]–[18]; for review, see [19]; for recent discussion, see [13]). Suppression has been conceptualised as a ‘switching off’ of vision from one eye that arises in binocular viewing conditions only; as soon as viewing becomes monocular (e.g. when the fellow eye is closed), suppression disappears and objects that were previously invisible suddenly appear [2]. While suppression is believed to help deal with problems like diplopia and confusion, it is also thought to have potentially serious consequences if it persists. In individuals with both amblyopia and strabismus, for example, suppression is classically believed to act as the conduit linking amblyopia and strabismus (e.g. [20], [21]). Specifically, chronic, long-standing suppression (e.g. from a constant, unilateral strabismus) is believed to lead to amblyopia [11], [12] [20], [21]. The evidence offered in support of this claim includes the finding that suppression may be deeper in individuals with deeper levels of amblyopia [22]–[24] (but see [25], [26]). However, the evidence that strabismic suppression and amblyopia are causally linked is weak and the prevalence, role and indeed even the very existence of suppression in individuals with strabismus and amblyopia are subject to a number of challenges, which we now consider.

Firstly, from a clinical perspective, suppression is extremely difficult to measure and the results of testing are inherently variable. The depth and extent of suppression are critically dependent upon how it is evaluated [14], [18], [19]. Frequently, for example, suppression may be evident using one measurement technique but absent when another method is employed. This raises the possibility that suppression is an artefact of testing rather than a genuine clinical phenomenon [13], [14], [27]. Secondly, whereas suppression was once thought of as a complete ‘switching off’ of vision from one eye, recent studies that have modelled psychophysical data gathered in individuals with amblyopia and strabismus are seriously challenging our ideas about suppression. For example, there is considerable, ongoing research attention devoted to the question of whether or how suppression in individuals with amblyopia and strabismus differs from dichoptic masking in visually normal persons [28]. Although a full consensus has not so far been reached, there is now a substantial body of research evidence that suggest that the results in strabismic amblyopes can be modelled using a mechanism that is quantitatively but not qualitatively different to the model that explains dichoptic masking results in normals [29], [30]. We return to this topic in the discussion.

Why is it important to understand binocular interaction in individuals with these developmental disorders of vision? Firstly, a comparison between normal and subnormal binocularity can provide insights to how normal binocular vision works. Secondly, our understanding of the nature of the visual deficit experienced by individuals with these conditions and the identification of optimum treatment paradigms could be greatly enhanced if we can understand the underlying mechanisms involved in creating the disorder(s). For example, there is now considerable interest in the possibility that residual binocularity in individuals with amblyopia and strabismus [31]–[38] may offer new approaches upon which to base treatment [39]–[41].

**Figure 1:**
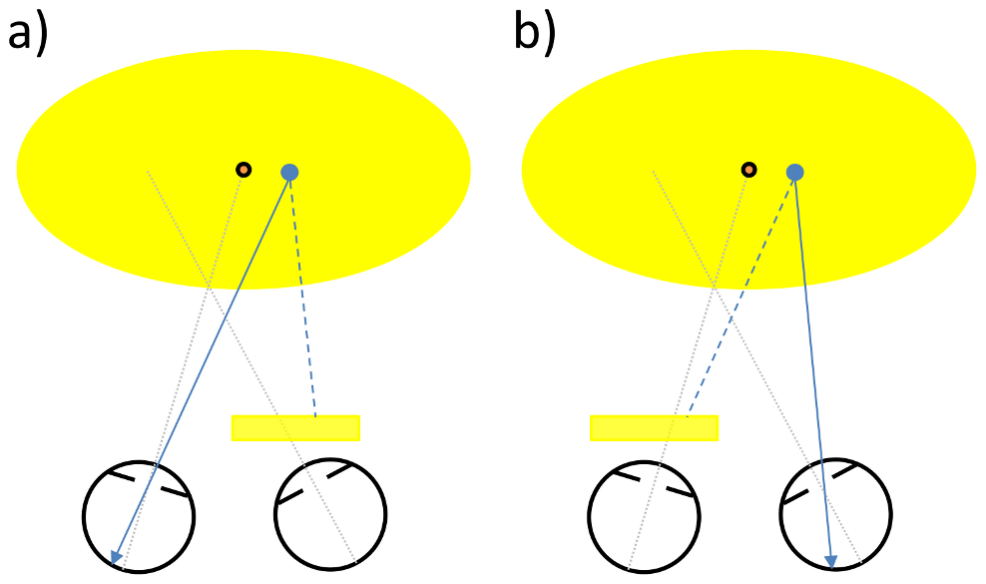
Schematic arrangment of viewing conditions for a hypothetical strabismic participant with a right eye (RE) esotropia. a) Yellow filter is placed in front of the strabismic RE. This makes the blue stimulus to be detected invisible to the RE. Hence sensitivity to the blue stimulus reflects LE (fixating eye) performance only. Dissociation between the eyes is minimal because the RE sees all except the blue stimulus. There is minimal dissociation because both eyes are presented with yellow background. b) Identical to a) except that yellow filter is in front of the non-strabismic LE. Sensitivity to the blue stimulus is now determined by the RE alone, albeit at a greater retinal eccentricity as the RE remains in its habitual motor position. When no yellow filter is used (not shown), the blue stimulus is potentially visible to both eyes and thus the detection threshold reflects sensitivity in habitual viewing (i.e. with both eyes open, blue stimulus available to both eyes and the deviating eye in its anomalous motor position). These three viewing conditions enable the monocular- and binocular-sensitivities to be determined and compared with the eyes in identical positions for the three conditions. Thus an examination of the extent, if any, to which the deviating eye contributes to sensitivity in habitual viewing is possible.

In recent work [13] we have assessed whether the deviating eye in individuals with strabismic amblyopia is suppressed. To address particular difficulties associated with the assessment and measurement of suppression, we employed an experimental method to test the sensitivity of the deviating eye in its habitual motor position in conditions of minimal rivalry (Figure 1). Surprisingly, and contrary to clinical dogma [11], [12], we found very little evidence of suppression in our strabismic amblyopes. The failure to find convincing evidence of suppression suggests that the deviating, affected eye’s signal is, for at least some kinds of stimulation, available at a conscious level. This availability, however, is not in itself a guarantee that the deviating eye contributes usefully in binocular viewing. The present study was designed to examine the extent to which the deviating eye makes a demonstrable contribution when the eyes occupy their habitual position, however anomalous that might be. This topic has relevance not only to the question of functional consequences of living with strabismic amblyopia but it is also relevant to wider questions about the prevalence and role of suppression, and about the nature of the relationship between strabismus and amblyopia.

## Results

**Figure 2:**
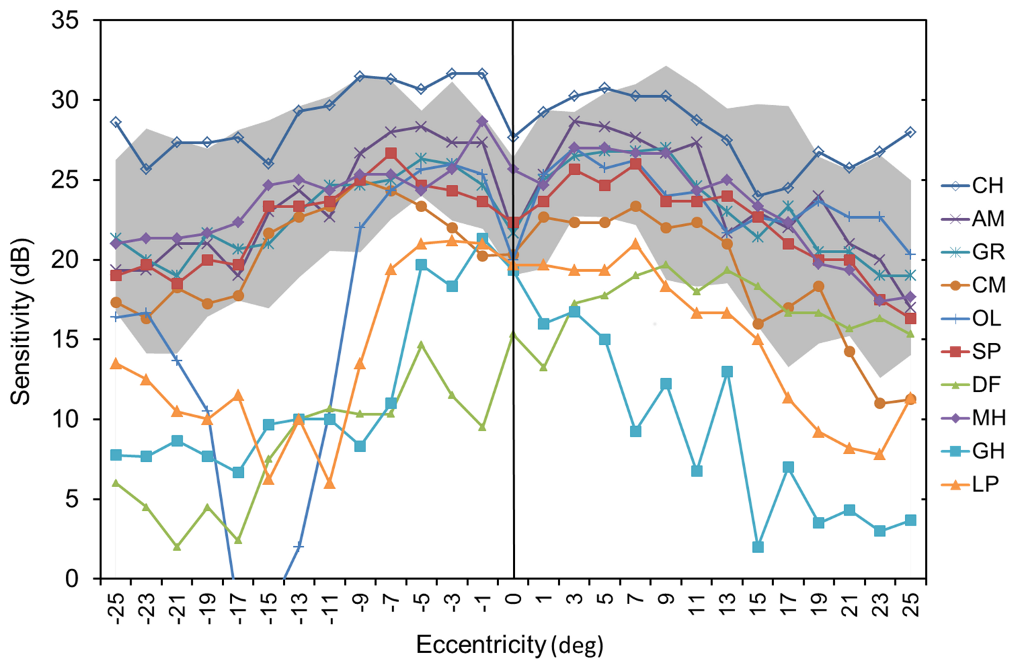
Data from individuals with strabismic amblyopia in binocular viewing (both eyes open and potentially able to detect the blue stimulus and non-dominant eye in its habitual, deviated position) compared with the range of sensitivities found in the equivalent viewing condition in the visually normal controls (denoted by the shaded zone). ‘Sensitivity’ (y-axis) refers to the ability to detect the blue stimulus against the yellow background. Sensitivity is measured in decibels (dB) and, consistent with standard, white-on-white perimetry, a higher sensitivity indicates better performance. Error bars represent ±1 standard deviation of the mean. Clinical details for strabismic amblyopes are provided in Table 1.

The purpose of this study was not to compare visual field sensitivity in normals and strabismic amblyopes under binocular viewing conditions. However, to put the performance of our amblyopes participants on this task in context, we start by showing how performance with both eyes open (i.e. in their habitual motor position) in our strabismic amblyopes compared to our visual normals. Figure 2 shows sensitivities in the central 50 degrees along the horizontal midline for each of our strabismic amblyopes relative to the performance range exhibited by our visual normals. The majority of amblyopes exhibit binocular sensitivities on this task that overlap with visual normals, although there are some notable exceptions (participants GH, LP, DF & OL; Table 1 & Figure 2]. Participants DF, GH & LP are amongst the oldest of our participants and yellowing of the crystalline lens with age will account for at least part of their sensitivity loss on account of the greater absorption of short wavelength light [42]. The fact that most participants exhibited sensitivity within the normal range is consistent with the view that amblyopia does not lead to a dramatic loss of sensitivity on perimetric tasks ([43], [44]), although most previous studies arriving at this conclusion used white on white tasks whereas we used a blue-on-yellow task.

**Table 1:** Clinical details of individuals with strabismic amblyopia.

Initials	Age	HabRVA	HabLVA	Domeye	OptimalRE Rx	OptimalLE Rx	OptimalVA of NDE	OptimalVA of DE	Nearangle	Bagolini	EF	Stereoacuity	Habitual Rx
AM	21	0.30	−0.10	L	+1.00/−0.75×15	+1.00DS	0.30	−0.10	6Δ RSOT	R Sup	1°N	300″	Optimal
CH	20	−0.10	0.48	R	Plano	+4.50DS	0.40	−0.10	6ΔLXOT	L Sup	1.5°T	600″	None
CM	26	−0.02	0.50	R	+5.75/−1.75×110	+6.00/−2.00×68	0.50	−0.02	10ΔLSOT	L Sup	2.5°N	Negative	Optimal
DF	41	−0.20	0.20	R	+2.00DS	+3.75/−0.25×65	0.18	−0.20	4ΔLSOT	No sup	0.5°N	600″	RE: +2.00 DS, LE: +2.00 DS
GH	54	−0.10	0.58	R	+1.25/−0.25×90	+4.75/−0.25×80	0.54	−0.10	14ΔLSOT	L Sup	1.5°N	Negative	RE: +0.50 DS, LE: +4.00 DS
GR	23	0.20	−0.10	L	+6.00/−3.25×85	+4.00/−2.00×75	0.20	−0.10	4ΔRXOT	No sup	0.5°T	300″	Optimal
LP	56	0.80	0.18	L	+2.00DS	+3.50/−3.00×45	0.80	0.18	10ΔRXOT	R Sup	2°T	Negative	Optimal
MH	46	0.30	0.00	L	+3.00DS	+2.75DS	0.30	0.00	10ΔRSOT	R Sup	2°N	Negative	Optimal
OL	28	0.84	0.00	L	+4.25/−3.50×170	+1.50/−1.25×175	0.80	0.00	45ΔRXOT	R Sup	2.5°T	Negative	RE: +2.75/−3.50×170,LE: 0.00/−1.25×175
SP	36	0.42	0.00	L	+1.75/−0.50×5	−0.50/−0.25×10	0.38	−0.06	10ΔRSOT	R Sup	2°T	Negative	Optimal

Monocular visual acuity (VA, logMAR) are presented for left (L) and right (R) eyes under both habitual and optimal refractive conditions for the non-dominant eye (NDE) and dominant eye (DE). The refractive correction worn habitually by each participant is also shown as Optimal (same as optimal refraction), None (no correction worn) or detailed in other cases. Oculomotor status and measurements at near viewing are presented and stereoacuity is given in seconds of arc (negative indicates worse than 600″) as measured with the Frisby stereo acuity test. Eccentric fixation (EF) was measured in strabismic amblyopes using an inter-ocular afterimage transfer method and the results are presented in degrees nasal (N) or degrees temporal (T). In visual normals, ocular dominance was determined with the Kay-pictures Dominant Eye Test (www.kaypictures.co.uk/dominant.html) and was classified as the eye that was used for sighting on at least two of the three presentations. The presence of abnormal retinal correspondence (ARC) was inferred from the results of the Bagolini lens test. On this basis only two participants (DF & GR) were diagnosed as displaying ARC. All four exotropes are primary rather than consecutive exotropes. Abbreviations: AE = Amblyopic Eye, FE = Fellow Eye, Dom = Dominant Eye, XOT = Exotropia, SOT = Esotropia, Sup = suppression, DS = Dioptre Sphere, Δ = Prism Dioptres.

**Figure 3:**
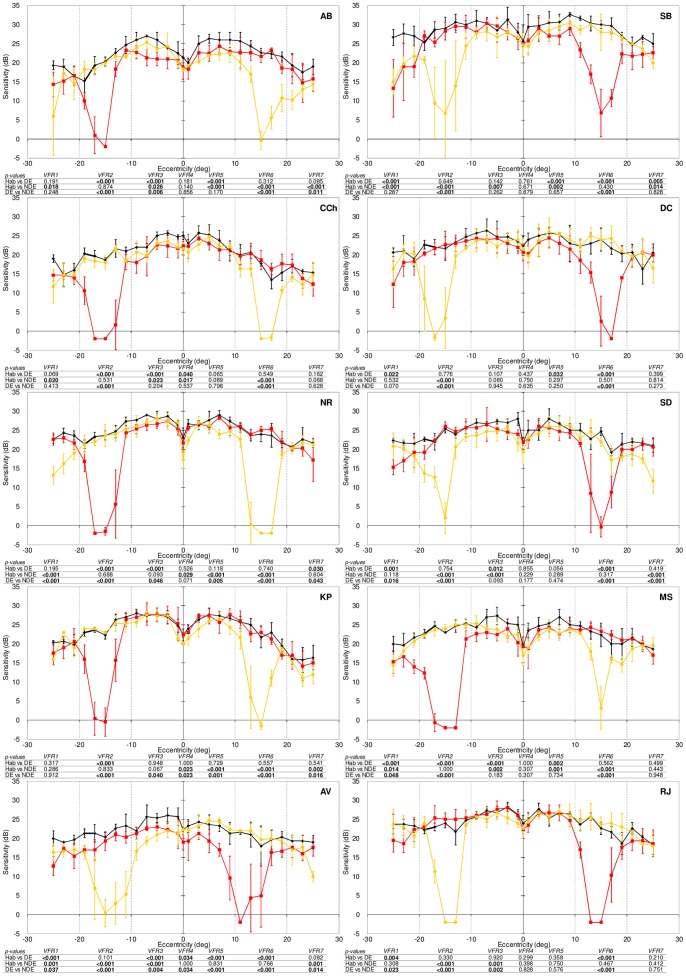
Results from visual normals. ‘Sensitivity’ (y-axis) refers to the ability to detect the blue stimulus against the yellow background. Sensitivity is measured in decibels (dB) and, consistent with standard, white-on-white perimetry, a higher sensitivity indicates better performance. Monocular sensitivities of the non-dominant (NDE, yellow) and dominant (DE, red) eyes are plotted for comparison against sensitivity during habitual viewing (black) when the blue stimulus to be detected was presented to both eyes. Error bars represent ±1 standard deviation of the mean. Negative values on the eccentricity axis correspond to visual field locations to the left of the straight-ahead position. P-values are from the regression analysis within each region separated by the vertical dotted lines. For each visual field region, p-values are displayed for a comparison of habitual versus dominant-eye sensitivity (Hab vs. DE), habitual versus non-dominant eye sensitivity (Hab vs. NDE) and dominant versus non-dominant eye sensitivity (DE vs. NDE) in that region. P-values in bold are statistically significant using a criterion of p<0.05.

**Figure 4:**
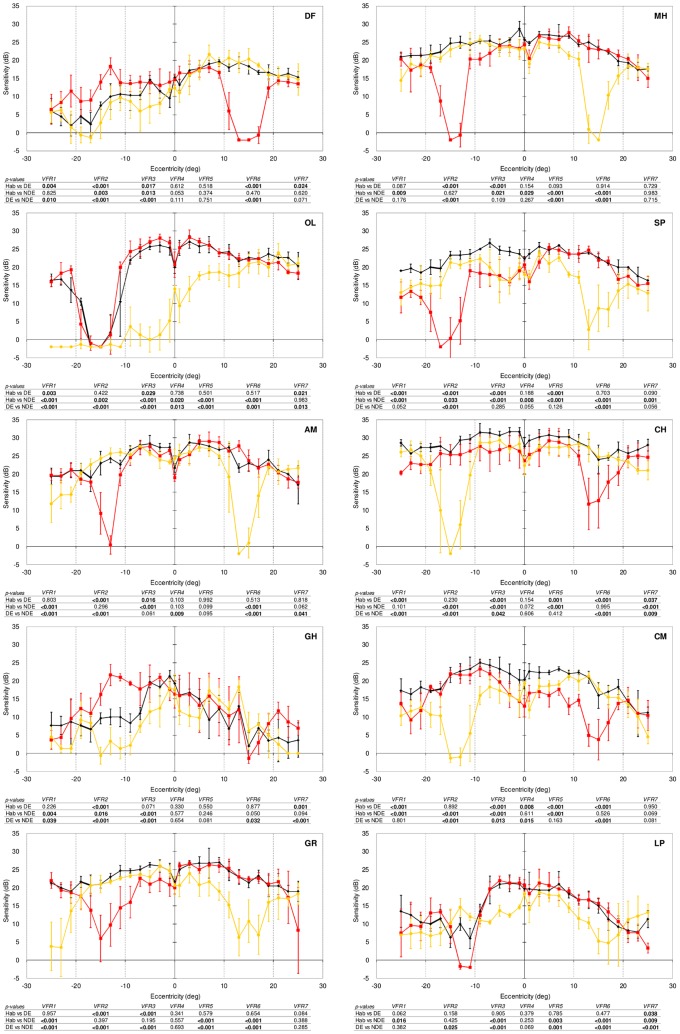
Similar to Figure 3 except that results are shown for individuals with strabismic amblyopia.

The main purpose of this study was to examine how sensitivity in strabismic amblyopes compares to the sensitivities of the dominant (fixating) and non-dominant (deviating) eyes so as to ascertain the contribution, if any, of the weaker eye. Using the approach outlined in the ‘Data Analysis’ section we searched for evidence that the non-dominant eye contributes, either positively or negatively, to sensitivity under binocular viewing conditions. First, we examine the results in visual normals (Figure 3) so as to be able to contextualise the results in our participants with strabismic amblyopia (Figure 4).

### Results in Visual Normals

**Figure 5:**
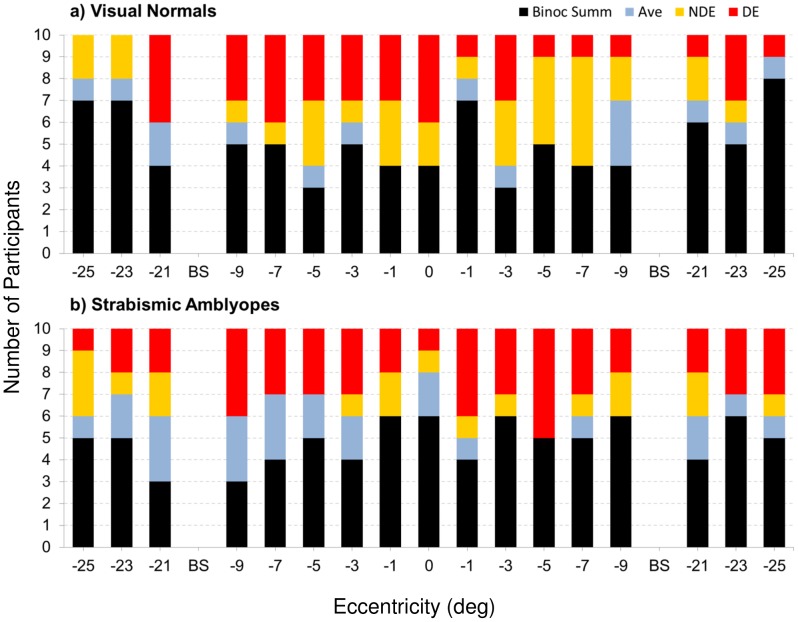
Summary of results obtained in visual normals (top, (a)) and in strabismic amblyopes (bottom, (b)). To account for the fact that in normals and strabismic amblyopes, the dominant eye may be the right or left eye, the results for some participants have been flipped so that the dominant eye is always the right eye, and the non-dominant eye is always the left eye. Locations corresponding to the blind spot (BS) in each eye were excluded from this analysis and are thus shown as gaps in the figure. Each bar corresponds to a visual field location in degrees to the left or right of the straight ahead position. The proportion of each bar that is black in colour indicates the proportion of normals (a) or strabismic amblyopes (b) for whom the binocular summation (Binoc Summ) model provided the closest estimate of binocular sensitivity actually exhibited at that field location. The proportion of individuals for whom the average monocular sensitivity is closest to binocular sensitivity is coded in blue (Ave), and red and yellow (NDE) colours represent the proportions where, respectively, the dominant (DE) and non-dominant (NDE) eye sensitivity is closest to the binocular sensitivity actually exhibited.

Figure 3 shows monocular and binocular sensitivities for our ten visual normals. The equation
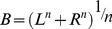
(1)combines the sensitivities for the left (L) and right (R) eyes to produce a predicted binocular (B) sensitivity [45]–[47]. The value of the exponent (n) in this equation dictates the extent to which binocular sensitivity differs from the sensitivity in monocular viewing; for example, when n is 2, binocular sensitivity exceeds monocular sensitivities by a factor of 1.4 (classical binocular summation), whereas higher values of n reflect smaller and smaller amounts of summation (see ‘Data Analysis’ of *Methods*). We established the value of n separately for each visually normal participant so that the modelled binocular sensitivity using equation [1] was on average within 1 dB, across the field locations tested excluding either blind spot, of the binocular sensitivity actually observed (Figure 3). The values of n for our visual normals ranged from 3.6 to 9.2, with an average value for the exponent (n) of 5.9 in Equation [1]. To characterise the relationship between monocular and binocular performance we compared the binocular sensitivity actually exhibited at all individual field locations in all normals (black curves/symbols in Figure 3) with (i) the binocular sensitivity predicted using n = 5.9 in Equation [1] (Binocular summation model), (ii) the sensitivity actually exhibited by the dominant eye, (iii) the sensitivity actually exhibited by the non-dominant eye, and (iv) the average of the monocular sensitivities. This allowed us to identify in each participant, at each field location, whether binocular sensitivity reflects binocular summation of the right and left eye sensitivities, whether it reflects an average of these monocular sensitivities or whether sensitivity under binocular conditions corresponds to the sensitivity of the dominant or non-dominant eyes. Figure 5(a) shows a summary plot of the results of this comparison in our group of visual normals. In around half (50.6%) of field locations, the binocular summation model provided the best description of the binocular sensitivities actually exhibited (black colour in Figure 5a). The proportions of field locations for which the dominant and non-dominant eyes explained sensitivity under binocular conditions were each around 20% (21.2% dominant, Figure 5a; red; 19.4% non-dominant: yellow). In the remaining field locations (8.8%, Figure 5a, blue), binocular sensitivity corresponded most closely to the average of the monocular sensitivities at that location.

The binocular summation model explained performance in the majority of field locations in seven of the ten visual normals (participants AB, AV, CCh, MS, NR, SB & SD). In two normals (participants KP & RJ), dominant eye sensitivity accounted for binocular performance to a greater extent that the other models. In the remaining visual normal (participant DC), binocular sensitivity most frequently reflected sensitivity of the non-dominant eye. We now compare this pattern of results with results of an equivalent analysis in our participants with strabismic amblyopia.

### Results in Participants with Strabismic Amblyopia

To facilitate direct comparison with the results in our visual normals, we again used an exponent (n) value of 5.9 in Equation [1]. As in normals, the sensitivities exhibited under binocular viewing conditions at all field locations in our strabismic amblyopes (Figure 4) were compared with the four models (binocular summation, average of monocular sensitivities, dominant and non-dominant eye sensitivities) outlined above and described in more detail later in the ‘Data Analysis’ section. Figure 5(b) shows a summary plot of the results across our group of strabismic amblyopes. As in our visual normals, in around half (48.2%) of field locations, the binocular summation model provided the best description of the binocular sensitivities actually exhibited (black, Figure 5b). The proportions of field locations for which the dominant eye explained sensitivity under binocular conditions was 27.1% (Figure 5b: red; compared to 21.2% in normals). In contrast to visual normals, only 10.6% of field locations displayed sensitivity under binocular conditions that was best explained by non-dominant eye sensitivity (Figure 5b: yellow, compared to 19.4% in normals). In the remaining 14.1% of field locations, binocular sensitivity corresponded most closely to the average of the monocular sensitivities at that location (compared to 8.8% in normals, Figure 5b, blue).

The binocular summation model explained performance in the majority of field locations in six of the ten strabismic amblyopes (participants CH, CM, GH, GR, MH & SP). In a further three participants with strabismic amblyopia (AM, LP & OL), dominant eye sensitivity accounted for binocular performance to a greater extent than the other models. In the remaining strabismic amblyope (participant DF), binocular sensitivity mainly reflected the average of the monocular sensitivities.

Overall, the results in strabismic amblyopes (Figure 5(b)) reveal a similar occurrence of summation compared to visual normals (Figure 5(a), 48.2% amblyopes versus 50.6% normals). Strabismic amblyopes exhibited a smaller incidence of the non-dominant eye explaining binocular sensitivity (10.6% versus 19.4% in normals). This is offset by an increased incidence of the dominant eye explaining binocular sensitivity (27.1% and 21.2% in amblyopes and normals, respectively) and by a greater incidence of binocular sensitivity reflecting the average of the monocular sensitivities in amblyopes (14.1%) compared to normals (8.8%). The proportion of visual field locations in which the binocular summation model best explained performance described here does indeed depend on the value of the exponent (n) chosen. Results are presented here using a value derived from the average performance of the visual normals in the study. The analysis was repeated with values of the exponent (n) between 2 and 7 and results are presented in Table 2. Whilst the proportion of field locations best described by the binocular summation model change as a function of n, the similarity in the profile of results for each of the two groups remains (Table 2).

**Table 2:** Outcomes of modelling results at each location as a function of exponent (n).

	Amblyopes				Normals			
N	BinocSumm	DE	NDE	Ave	BinocSumm	DE	NDE	Ave
2	15.3%	45.9%	24.7%	14.1%	7.1%	43.5%	40.6%	8.8%
3	28.9%	38.8%	18.2%	14.1%	23.6%	33.5%	34.1%	8.8%
4	40.0%	32.4%	13.5%	14.1%	36.5%	26.5%	28.2%	8.8%
5	43.0%	29.4%	13.5%	14.1%	45.4%	22.9%	22.9%	8.8%
5.9	48.2%	27.1%	10.6%	14.1%	50.6%	21.2%	19.4%	8.8%
7	54.2%	22.9%	8.8%	14.1%	58.3%	17.6%	15.3%	8.8%

The proportion of visual field locations best described by each of the four models for strabismic amblyopic and visual normal groups as a function of the exponent(n) used in the Binocular Summation modelling using equation [1]. The numbers are the percentages of individual locations over the ten participants in each group in which the measured binocular visual field sensitivity was closest to the modelled Binocular summation (BinocSumm), the sensitivity in the Dominant Eye (DE), the sensitivity in the Non-dominant Eye (NDE) or the average of the sensitivities of the dominant and non-dominant eyes (Ave).

## Discussion

If the deviating eye in strabismic amblyopes is suppressed in habitual viewing (i.e. with both eyes open and the deviating eye in its habitual, anomalous motor position), the absence of the deviating eye’s signal should mean that two eyes are not better than one and that, except in the blind-spot of the fixating eye, performance with both eyes open should simply reflect performance of the better eye. In recent work using a similar testing approach and paradigm with the same group of strabismic amblyopes we found little evidence that the deviating eye’s signal was suppressed (see below, also [13]). However, the absence of suppression does not necessarily mean that the deviating eye impacts upon the sensitivity exhibited in binocular viewing. Our results show clear evidence that the deviating eye in strabismic amblyopes can be responsible for enhancing sensitivity in binocular- versus dominant-eye-only viewing; summation was evident in around half of non-blind spot field locations (49.2%, compared to 50.2% in visual normals). In addition, we find evidence that even in areas of the visual field that do not correspond to the blind spot of the dominant, fixating eye, sensitivity under binocular conditions can reflect the sensitivity of the non-deviating eye (10.6% of non blind-spot locations tested). These patterns of a positive deviating-eye contribution are not ubiquitous across the visual field regions that we tested and they were not evident in all of our strabismic amblyopes. Indeed, in three of our ten participants (AM, LP, OL, Figure 3), sensitivity in binocular viewing reflected the sensitivity of the dominant eye in a majority of the field locations we tested. Overall, however, our results demonstrate strong evidence that the deviating eye of most individuals with strabismic amblyopes contributes in a positive fashion in binocular viewing. The availability of the deviating eye’s signal could of course mean that binocular sensitivity is lower than one or other of the monocular sensitivities. This could reflect a negative contribution from the deviating eye. We did find evidence of a negative contribution from the deviating eye but it was extremely limited; in only one participant (DF, Figure 3) did binocular sensitivity predominantly reflect an average of monocular sensitivities, and across all participants, the proportion of field locations for which average monocular sensitivity corresponded to the actual binocular sensitivity was only 14.1% (compared to 8.8% in visual normals). Thus we find that the deviating eye in individuals with strabismic amblyopia is not suppressed, indeed in the majority of individuals, and at the majority of field locations we tested, it makes a positive contribution to the sensitivity exhibited under binocular viewing conditions.

Although the focus of this study was on the contribution by the non-dominant eye in habitual viewing, our results are relevant to more general discussions about binocular interaction and suppression in individuals with amblyopia and/or strabismus. One view of suppression is that it is delivered via visual mechanisms that are structurally and functionally different from normal processing mechanisms. However, the view that appears to be emerging from recent psychophysical and modelling studies of humans with strabismus and amblyopia is that the system remains structurally binocular, but it is the properties of binocular combination that are abnormal. For example, the results in strabismic amblyopia can be explained by attenuating the signal from the weaker eye and increasing the ‘noise’ in the amblyopic eye [29]. Baker et al.’s model of strabismic amblyopia [29] contains intact stages of interocular suppression and binocular summation and other evidence from the same group [30] also suggests that binocular summation mechanisms are intact. Similarly, the deficits in anisometropic amblyopia can be modelled by signal attenuation and interocular inhibition [48]. The idea that the visual system in strabismic amblyopes may be structurally binocular but, under typical viewing conditions, functionally monocular is certainly different to long-held clinical views about the nature of suppression [2]. The critical importance of the stimuli presented to the two eyes when examining suppression is illustrated by Huang et al [49] who reported a weak masking effect in the presence of dichoptic full-field luminance modulation but a much stronger masking effect when dichoptic contrast modulation of a noise texture was employed. This indicates the contrast-dependent nature of suppression. Considering Huang et al’s [49] results, one interpretation of our findings is that they may reflect a peculiar set of viewing conditions which differ from habitual viewing to the extent that they can reveal a residual, but usually hidden, binocularity in strabismic amblyopes.

Our results share some similarities with results obtained in patients with glaucomatous visual field damage [50]. In that study, binocular visual field sensitivity to white-on-white targets was predicted from monocular visual field results in a large sample of patients with varying degrees of visual field loss in one or both eyes. Nelson-Quigg et al [50] used a similar modelling approach to the approach we employed here. Glaucoma is, of course, not the same as strabismic amblyopia; the latter is a unilateral condition whereas glaucoma is bilateral, although the two eyes are frequently affected to different extents [51]. Nevertheless, the task of predicting binocular sensitivity from monocular sensitivities in glaucoma patients and strabismic amblyopes shares similarities because of the likelihood that differential inter-ocular sensitivities exist. Nelson-Quigg [50] found that the ‘binocular summation’ and ‘best eye’ models performed similarly well and they both out-performed the average monocular sensitivities model. Our results are in many respects consistent with these findings.

As earlier indicated, in recent work we examined whether this same group of individuals with strabismic amblyopia exhibited suppression. We found little evidence to support the view that, when presented to the deviating eye, the target was suppressed; only three of these same ten participants showed any evidence for suppression and when it existed it was limited in extent and small in magnitude, typically less than 5 dB. We have previously discussed [13] how the methodology used to examine suppression status in individuals with strabismus, amblyopia or both is crucially important to the outcome ([13], [14,] [19], [52]–[54]) and about how the requirements of the task we used (which is identical to that employed in the present study) may have influenced our finding of limited suppression. Much of that discussion [13] is also relevant here because testing conditions that are likely to give rise to suppression (e.g. stimuli presented for long durations, [55]) are likely to be those in which a non-dominant eye contribution is difficult or impossible to reveal. We now ask whether there is something about our choice of task that may have made the deviating eye likely to exhibit a contribution in a way that would not have been evident if the task was different. In other words, how likely are the results from this experiment to generalise to tasks that more closely mimic habitual viewing?

The first factor to consider is that the task was to detect a blue stimulus on a yellow background. If blue-on-yellow sensitivity is spared in strabismic amblyopia, perhaps this could serve as an explanation for our finding of a substantial contribution by the non-dominant eye. We think this is unlikely because there is no evidence that sensitivity to chromatically-defined stimuli is affected in amblyopia to a greater or lesser extent than sensitivity to achromatic, luminance-defined information [56]–[58]. Secondly, our task represents a straight-forward case of detecting a point of light against a uniform background. It is certainly possible that the absence of any form in the field could explain the absence of any clinically significant suppression amongst our participants [13] and the availability of the deviating eye’s signal would have at least created the conditions in which the deviating was capable of contributing in the manner we observed. Whether a similar or, in fact, any contribution would be evident if we had employed a more complex task (e.g. resolution of target rather than simple detection) or if we presented our stimulus on a background containing form is not clear. This represents a potentially very useful avenue to consider for future experiments. Other aspects of our task that may have predisposed individuals with strabismic amblyopia to show evidence for a contribution from their weaker eye include the duration and temporal profile of the stimulus to be detected (discussed in detail in [13]). Thus, in the same way that the absence or presence/magnitude of suppression appears critically dependent on the method used to dissociate the eyes ([13], [14], [18], [19]) and upon the task employed, any evidence for contribution by the non-dominant eye in individuals with strabismus, amblyopia or both may also depend critically upon the methodology used and the specifics of the task used to search for this contribution.

The average value of the exponent (n) in Equation 1 for our visually normal participants was close to 6. An exponent of 2 is typical for binocular contrast detection and other binocular visual tasks [45]–[47]. Thus our visual normals did not exhibit binocular summation on the task to the extent that may have been evident if a different task or stimulus arrangement had been employed. For example, Wood et al [59] have shown that binocular summation shows regional variations across the visual field and is also critically dependent on the target size. Had we chosen a task on which binocular summation was of greater magnitude in visual normals, it is possible that we would have found even greater evidence for non-dominant eye contribution in strabismic amblyopes. Equally however, a different task in which greater binocular summation is evident in visual normals may have uncovered major limitations in the extent to which the non-dominant eye contributes to performance in habitual viewing. Further work is clearly need to resolve this issue.

If our findings are robust in suggesting that the non-dominant eye makes a useful contribution in habitual viewing, this would be consistent with evidence from other studies that two eyes are better than the better alone in individuals with naturally-occurring disrupted binocular vision. In a gait and obstacle crossing task we found that the clearance allowed over the obstacle increased when the affected eye was covered [60]. This suggests these participants were not behaving like visual normals who had closed one eye. Revealing a contribution from the non-dominant eye is of interest but understanding the mechanism by which it contributes represents a further challenge. In the case of the gait/obstacle avoidance task, for example, it could be that the non-dominant eye’s contribution arises simply because it increases the overall size of the field, and/or that it enables concordance in optic-flow patterns to be used as a cue to aid task performance. Another possibility is that the contribution from the non-dominant eye is in the form of disparity processing, at a level that is perhaps too coarse to be able to reveal with standard clinical tests. There is evidence that at least some individuals with strabismus/strabismic amblyopia have residual disparity processing capabilities [30], [32]–[35]. It appears that in some individuals binocular interactions (e.g. as revealed by summation) only become apparent when the signal strength to the better eye is reduced appropriately (e.g. [33]). Furthermore, there is now a considerable volume of research in the area of amblyopia treatment which aims to tap and then strengthen residual binocular mechanisms [36]–[41]. We are not certain about the mechanism via which the non-dominant eye is contributing in the present task but we can rule out increased visual field size since assessment was confined to the central fifty degrees of visual space, and residual disparity processing can also be ruled out since the task of detecting our stimulus is not disparity-dependent.

The possible implications of our results extend beyond understanding how strabismic amblyopia impacts upon binocular performance. Firstly, demonstration of a contribution from the non-dominant eye in individuals with strabismic amblyopia provides a more robust test of binocular co-operation compared to evidence that suppression is absent or limited. This is because while the absence of suppression indicates that the non-dominant eye’s signal is available, evidence of non-dominant eye contribution through, for example binocular summation, indicates how and to what extent that signal is useful. Secondly, the results we present here, together with previous results [13] showing a limited role for suppression, suggest that we may be underestimating the contribution and usefulness of the deviating eye in strabismic amblyopes. Strabismus is believed to represent one of the major causes of human amblyopia [11], [12], and although suppression is often invoked, the mechanism which links strabismus and amblyopia is very poorly understood. The next step is to establish whether the results we have obtained with this group of strabismic amblyopes (minimal suppression, non-dominant eye contribution) are peculiar to the task and/or methodology we have employed. If it turns out that these results hold true in conditions featuring more complex visual tasks that more closely mimic habitual viewing conditions, the status of the deviating eye in strabismic amblyopes as chronically suppressed and therefore making little contribution in the central field may need to be re-evaluated. This is now the subject of work in progress.

## Methods

### Ethics Statement

The tenets of Declaration of Helsinki were followed and the study had approval of the University of Bradford Ethics Committee, with written informed consent being obtained from all participants prior to their participation.

### Participants

A total of 10 adults with strabismic amblyopia took part. Prior to participation all subjects underwent full eye examination and binocular vision assessment. Clinical details for each study participant are presented in Table 1. All of our participants exhibited a manifest strabismus on cover/uncover testing. Ten visually normal control subjects also participated in the study. The individuals who participated in the study (normals & individuals with strabismic amblyopia) were the same individuals as in our previous, related study [13].

### Protocol

Our experimental protocol has been described elsewhere [13]. Briefly, using the Humphrey Visual Field Analyzer (HFA, model 745i, Carl Zeiss Group), we assessed sensitivity to a narrow-band, blue light stimulus with a peak wavelength of 440 nm (Goldmann size III target, 0.43 degrees) presented on a 100 cd/m^2^ broadband (500–700 nm) yellow background with a stimulus duration of 200 msec. We employed this blue-on-yellow detection task because it provided a straight-forward means for assessment of the deviating eye’s sensitivity in its habitual motor position using the HFA instrument. Using a yellow filter (010 medium yellow, Lee filters, www.leefilters.com/lighting/products/finder/act:colourdetails/colourRef:C4630710C3E644) over one eye provided viewing conditions in which each eye sees the same, apart from the blue light stimulus which is only seen by the eye without the filter; the absorption characteristics of the filter ensured that the blue stimulus was invisible to the eye with the filter. This form of dichoptic viewing was favoured as it allowed investigation of the thresholds from one eye with no effect on motor position; the filter permitted the subject to view inside the bowl of the HFA with both eyes open as normal with the non-dominant eye in its habitual, deviated position. Alternative dichoptic viewing arrangements with different methods of dissociation could potentially alter the habitual motor position of the eyes (e.g. mirror haploscopic methods) or introduce rivalrous conditions (e.g. red/green dissociation). We assessed sensitivity in this blue-on-yellow detection task in three different viewing conditions (see below).

The blue-on-yellow detection task that we employ is the same as in standard automated perimetry in which a higher sensitivity (measured in decibels (dB) indicates an ability to detect a dimmer blue stimulus. Sensitivity was assessed in the straight ahead position, and at two degree intervals from 1 to 25 degrees along a horizontal meridian on either side of the straight ahead position. In order to avoid the horizontal raphe, measurements were assessed two degrees above the horizontal midline. Each participant viewed the target through full aperture trial lenses, positioned in a lightweight trial frame, as determined by inputting their age and optimal distance refraction (Table 1) into the HFA software. Participants received standard perimetric instructions regarding the procedure to be followed using the HVFA. Specifically, they were asked to maintain fixation at the centre of the diamond target throughout and to press the button on the hand-held unit when a blue light was detected.

Fixation was monitored manually by the clinician using the video camera and by the eye-tracking device of the HVA and participants were reminded, as necessary, throughout the trial to fixate on the fixation target straight ahead.

The HFA full-threshold programme used estimates sensitivity at each stimulus location using a 4-2 dB staircase procedure: an initial crossing of threshold in 4 dB increments and a final crossing in 2 dB increments with the threshold designated as the last seen stimulus luminance. The short-term fluctuation option was enabled allowing the HFA to retest some stimulus locations either randomly or when the initial estimation of threshold deviated from that expected by comparison with neighbouring locations.

All participants undertook trial runs at the start of each data collection visit to ensure that the instructions were understood and that the participant was able to perform the task. The data from the trial runs were not included in the analysis. Data was collected for a total of six viewing conditions as part of a larger study, investigating not only the contribution of the deviating (i.e. non-dominant) eye (this study) but also suppression of the non-dominant eye [13]. One run of each viewing condition was collected on three occasions separated by between two and seven days. Each run lasted approximately nine minutes during which sensitivity was estimated at each of the 27 stimulus locations. Combining the three runs for each viewing condition produced a mean sensitivity at each location from between 3 and 6 estimates (median 4). In order to counter fatigue effects across participants, the testing order for the viewing conditions was randomised for each participant but held constant over each visit. The data from three of the viewing conditions are presented here (Figure 1).

1. Yellow filter over the dominant eye. This prevented the dominant eye from seeing the blue stimulus thus allowing the sensitivity of the non-dominant eye to be determined with dominant, fixating eye open and able to detect all other form. In this viewing condition the dominant eye maintains the ability to see the fixation target and thus was responsible for maintaining the habitual motor position of the two eyes. Importantly, therefore, data gathered in this condition reflected sensitivity of the non-dominant eye in its habitual, deviated position (Figure 1a).
2. Yellow filter over the non- dominant eye. This prevented the non- dominant eye from seeing the blue stimulus and thus allowed us to assess the sensitivity of the dominant eye (Figure 1b).
3. Habitual (i.e. binocular) viewing condition. In this condition, the yellow-filter was not used and, as a result, the blue stimulus was potentially visible to both eyes. The sensitivity determined under this viewing condition therefore reflects the sensitivity when the eyes are open and potentially both are contributing to the detection of stimulus (Figure 1c).

A group of 10 visually –normal control subjects (age range 20–44, mean 30.7 years) also took part in the study and attended for three visits of data collection in same viewing conditions (yellow filter over dominant eye, yellow filter over non-dominant eye and binocular sensitivity (i.e. no filter employed)).

### Data Analysis

It is important to point out that the motor status of the non-dominant eye is identical in all conditions described above (1, 2 and 3) allowing the data gathered in the three viewing conditions to be directly compared. As in our previous study [13], to compare the different viewing conditions for each participant, data were then grouped into seven regions of the horizontal meridian tested:

VFR1: −20 to −25 degrees from the straight ahead position

VFR2: −10 to −19.9 degrees from the straight ahead position

VFR3: −1 to −9.9 degrees from the straight ahead position

VFR4: −0.9 to 0.9 degrees from the straight ahead position

VFR5∶1 to 9.9 degrees from the straight ahead position

VFR6∶10 to 19.9 degrees from the straight ahead position

VFR7∶20 to 25 degrees from the straight ahead position

In the above notation, negative visual field locations are to the left of the straight ahead position and positive values are to the right.

A separate regression analysis (Stata version 9 1997, www.stata.com) was performed for each region, within each participant, to compare binocular sensitivity to the monocular sensitivities. The model used all available individual sensitivity values and the data from each condition was fitted with a second order polynomial function.

### Modelling the Results at each Visual Field Location

We predicted the binocular visual field sensitivity at each visual field location from the monocular sensitivities at that location using an approach that closely matches, but is not identical to, that used by Nelson-Quigg [50] in a sample of glaucoma patients. Using Equation [1] we determined the exponent (n) that, in visual normals, led to predictions of binocular of binocular sensitivity that were on average within 1 dB, across the field locations tested excluding either blind spot, of the binocular sensitivity actually exhibited. The average exponent (n) from Equation [1] [45]–[47] for the visual normals was 5.9. We then used the same exponent to make predictions of binocular sensitivity from monocular sensitivities in our strabismic amblyopes. For each participant, at each visual field location, we then compared the binocular sensitivity actually exhibited with (i) the predicted binocular sensitivity (binocular summation model). The binocular sensitivity actually exhibited was also compared with (ii) the average of the monocular sensitivities, and with the monocular sensitivity exhibited by (iii) dominant and (iv) non-dominant eyes. Depending on which of the four measures/predictions ((i), (ii), (iii) or (iv)) was closest to the binocular sensitivity actually exhibited at the visual field location, we labelled binocular sensitivity measures as representing instances of summation, averaging or matched monocular sensitivity (dominant or non-dominant). This classification formed the basis for the results presented in Figure 5.
